# Stomatognathic Diseases: State of the Art and Future Perspectives

**DOI:** 10.3390/jcm11216525

**Published:** 2022-11-03

**Authors:** Agostino Guida, Saman Warnakulasuriya

**Affiliations:** 1 U.O.C. Odontostomatologia, A.O.R.N. “A. Cardarelli”, 80131 Naples, Italy; 2 Faculty of Dentistry, Oral & Craniofacial Sciences, King’s College London, London SE1 9RT, UK

The World Health Organization (WHO) considers oral heath to be a key indicator of overall health, as it is linked to physical well-being and quality of life [1]. As the stomatognathic system consists of teeth, jaw bones, tongue, oral mucosa lining the mouth (including gingival tissues and lips), muscles involved in chewing and swallowing, salivary glands, and temporomandibular joints, there is a wide range of conditions that may undermine oral health: dental caries and periodontal (gum) disease leading to tooth loss, a wide range of oral mucosal diseases and oral cancer, xerostomia due to hypofunction of salivary glands, oro-facial pain, noma (necrotizing ulcerative stomatitis), and birth defects (such as cleft lip and palate).

The most common malignant condition that affects the oral cavity is oral squamous cell carcinoma (OSCC) [2], which arises from the mucosal lining of the oral cavity. On a global scale, more than 350,000 new incident cases of OSCC have been estimated, resulting in over 150,000 deaths in 2020 [3]. Despite clinical and therapeutic advances, the 5-year overall survival rate of oral cancer remains at 60%; the survival of patients with initial stages of OSCC stands at 80–90%, while it drops to under 50% for advanced-stage patients. It is apparent that the overall survival is low since most cases are diagnosed at advanced stages. Periodic surveillance and early detection by oral visual examination make up the foundation for effectively downsizing disease burden and possibly reducing the incidence of invasive cancer and related mortality [4]. In recent years, scientific literature also indicates how low-cost imaging techniques may represent a key strategy for non-invasive screening or the early detection of oral cancer [5,6,7,8,9,10,11].

Innovative therapeutic pathways which have proved themselves for cancers in other anatomical sites show limited results for OSCC. The lack of effectiveness of novel therapeutic approaches is partly due to the fact that the molecular biology of OSCC is not fully understood [12,13]. There are gaps in our knowledge of the natural history of OSCC, and not all oral potentially malignant disorders (OPMDs) undergo malignant transformation [14]; some remain stable, and some affected sites can revert back to health [15]. Moreover, OSCC can apparently develop from normal mucosa which may contain significant molecular aberrations that increase the likelihood of cancer [16,17,18]. Despite there being no consequentiality between OPMDs and OSCC, as well as between different grades of oral epithelial dysplasia, it has been reported that the risk of progression to oral cancer may increase in proportion to the severity of the dysplasia grade [19,20].

Some of the chronic oral inflammatory conditions are potentially malignant disorders too. Based on follow-up studies, there is now evidence of a low (1–2%) but significant risk of oral lichen planus (OLP) turning malignant with time [21,22,23], while the risk of the malignant transformation of chronic hyperplastic candidosis is still largely being debated [14]. Recent literature highlights potentially ground-breaking novelties for the diagnosis and follow-up of these chronic mucosal conditions, in terms of imaging and computer-assisted algorithm analyses [10,24]. 

The impact of tooth loss on global health is heavily linked to the resulting poor quality of life. The main cause of tooth loss in adults is periodontal disease. Periodontal disease is estimated to affect around one billion people worldwide (14% of the global adult population) [1]. Periodontitis is an inflammatory disease induced by an imbalance among bacterial virulence, microbiota dysbiosis, and a host defenses/inflammation [25]. Recently, scientific literature highlights the contribution of chronic inflammation to noncommunicable diseases, such as diabetes and obesity, as well as cardiovascular and neurological diseases. The link between these diseases seems to be low-grade inflammation (LGI) induced by pathogenic oral microbiota [26]; LGI causes a low-grade chronic systemic production of cytokines. LGI is a recognized risk factor for cardiovascular, cerebrovascular, and neurodegenerative diseases and cancer. Limited evidence also suggests that LGI increases the risk of insulin resistance and thus type 2 diabetes. LGI may be assessed through an evaluation of hematological (e.g., CRP, ESR, fibrinogen) or cell biomarkers (e.g., WBC and platelet counts), but this condition cannot be consistently defined or measured yet. Regarding the link between periodontitis and LGI, the most accredited hypothesis is that periodontitis may play a role in inducing/maintaining systemic LGI, and likewise LGI could promote periodontitis as an independent risk factor. Furthermore, the concept of LGI could explain the proved correlation among periodontitis and cardiovascular conditions or even degenerative neurologic conditions (e.g., Parkinson’s disease and Alzheimer’s disease).

As we briefly explored, despite their different origins, the pathologies of different organs comprising the stomatognathic system share common characteristics, the most important of which is that they may be heavily linked to common risk factors and general health conditions [1].

The Global Burden of Disease Study 2019 showed that oral diseases cause distress to around 3.5 billion people in total. Among these, the International Agency for Research on Cancer (IARC) evaluates that OSCC causes over 150,000 deaths each year. Furthermore, most oral conditions share risk factors with the main noncommunicable diseases (cardiovascular diseases, cancer, chronic respiratory diseases, and diabetes) and the scientific literature highlights the double link between periodontitis and conception/fertility [27]. The relationship between oral and general health is corroborated through shared risk factors such as tobacco use, alcohol consumption, and unhealthy diets unbalanced in carbohydrates. In addition to the aforementioned link between periodontitis and diabetes, the high consumption of sugars is seen as a common risk factor for diabetes, obesity, and dental caries. The WHO underlines how unhealthy lifestyles are alarmingly increasing at the global level. Furthermore, burning mouth syndrome (BMS) may be a probable link between mental health and oral health, and this peculiar oral condition is as a result of psychiatric distress in some cases. The aetiopathogenesis of this disease is complex and may be multifactorial, as local, systemic, and psychological factors are considered to be involved in generating symptoms. A recent publication points to the idea that enhanced pain perception in BMS could be linked to a higher frequency of white matter hyperintensities (WMHs) in the brain [28]. The literature indicates that psychotherapy and behavioral feedback may help to eliminate BMS symptoms [29]. 

With particular regard to frail older patients, the WHO recently included them in a resolution for global oral health [1], underlining how there is a strong link between oral diseases (OSCC, periodontal disease, and tooth decay) and conditions of social, cultural, and sanitary vulnerability, within the framework of an aging global population. Furthermore, conditions such as noma shall not be forgotten, as it mainly affects developing countries, especially children with poor health/quality of life or malnutrition.

Oral conditions are largely preventable and show dramatically better prognosis when treated at early stages. OSCC survival rates drop from 90–80% at stages I and II to around/less than 60–50% when treated at stages III-IV [9]. Primary (education on healthy lifestyle) and secondary (early diagnosis) prevention strategies are key factors for reaching success in reducing the burden of oral diseases which, according to the WHO, have to be integrated with primary health care. Furthermore, there is a necessity to gather data in order to determine oral health indicators and “best buys” and to implement cost-effective interventions for the population.

Researchers investigating oral health and disease from all round the globe must prosecute their part. *The Journal of Clinical Medicine*’s Special Issue, titled “Stomatognathic Diseases: State of the Art and Future Perspectives”, aims to contribute to this purpose, providing a platform for research papers on oral health with particular reference to its connection with systemic conditions.
